# Physicochemical Characteristics and Anticoagulant Activities of the Polysaccharides from Sea Cucumber *Pattalus mollis*

**DOI:** 10.3390/md17040198

**Published:** 2019-03-29

**Authors:** Wenqi Zheng, Lutan Zhou, Lisha Lin, Ying Cai, Huifang Sun, Longyan Zhao, Na Gao, Ronghua Yin, Jinhua Zhao

**Affiliations:** 1 School of Pharmaceutical Sciences, South-Central University for Nationalities, Wuhan 430074, China; zwq_scuec@126.com (W.Z.); zhaolongyan@mail.scuec.edu.cn (L.Z.); gn2008.happy@163.com (N.G.); 2 State Key Laboratory of Phytochemistry and Plant Resources in West China, Kunming Institute of Botany, Chinese Academy of Sciences, Kunming 650201, China; zhoulutan@mail.kib.ac.cn (L.Z.); linlisha@mail.kib.ac.cn (L.L.); caiying@mail.kib.ac.cn (Y.C.); sunhuifang@mail.kib.ac.cn (H.S.); 3 University of Chinese Academy of Sciences, Beijing 100049, China

**Keywords:** *Pattalus mollis*, fucosylated glycosaminoglycan, fucan sulfate, physicochemical characteristics, anticoagulant activities

## Abstract

Sulfated polysaccharides from sea cucumbers possess distinct chemical structure and various biological activities. Herein, three types of polysaccharides were isolated and purified from *Pattalus mollis*, and their structures and bioactivities were analyzed. The fucosylated glycosaminoglycan (PmFG) had a CS-like backbone composed of the repeating units of {-4-d-GlcA-β-1,3-d-GalNAc_4S6S_-β-1-}, and branches of a sulfated α-l-Fuc (including Fuc_2S4S_, Fuc_3S4S_ and Fuc_4S_ with a molar ratio of 2:2.5:1) linked to *O-3* of each d-GlcA. The fucan sulfate (PmFS) had a backbone consisting of a repetitively linked unit {-4-l-Fuc_2S_-α-1-}, and interestingly, every trisaccharide unit in its backbone was branched with a sulfated α-l-Fuc (Fuc_4S_ or Fuc_3S_ with a molar ratio of 4:1). Apart from the sulfated polysaccharides, two neutral glycans (PmNG-1 & -2) differing in molecular weight were also obtained and their structures were similar to animal glycogen. Anticoagulant assays indicated that PmFG and PmFS possessed strong APTT prolonging and intrinsic factor Xase inhibition activities, and the sulfated α-l-Fuc branches might contribute to the anticoagulant and anti-FXase activities of both PmFG and PmFS.

## 1. Introduction

Some sea cucumbers (*Echinodermata, Holothuroidea*) are popular tonic foods and traditional Chinese medicines in China for centuries [1]. Sulfated polysaccharides such as fucosylated glycosaminoglycan (FG) and fucan sulfate (FS) are the main components of polysaccharides extracted from sea cucumbers, and have attracted considerable attention due to their unique structures and extensive bioactivities [2,3,4,5,6].

FG is a distinct glycosaminoglycan (GAG) found exclusively so far in sea cucumbers [2]. Due to its multiple pharmacological activities such as antitumor, anti-thrombosis and anti-inflammation [7,8,9], and especially potent anticoagulation by inhibiting the intrinsic factor Xase complex (FXase) [8,10,11], FG has attracted increasing attention. It is generally agreed that FG possesses a chondroitin sulfate (CS)-like backbone which consists of the disaccharide repeating units {-4-d-GalNAcS-β-1,3-d-GlcA-β-1-}, and the backbone is branched with fucose sulfate (FucS) [2,10]. Factually, the structures of some FG, especially with various types of FucS branches, are still ill-defined [12,13,14,15,16,17]. For instance, early research showed that FG from *Stichopus japonicas* has a core structure of {-4-d-GalNAcS-β-1,3-d-GlcA-β-1-}, and mono-l-Fuc was linked to the each GlcA via α-1,3 linkage as the branch [15]. However, other researchers proposed that the Fuc branches also existed as a di- or tri-saccharide, and l-Fuc branches linked to GalNAc of backbone via α-1,4 or α-1,6 linkage [4,13,16].

Fucan sulfate (FS) is another type of sulfated polysaccharide from the body wall of sea cucumber. It was first reported in 1969, and it only comprises FucS [18]. Compared with fucoidans from brown alga, most FS from echinoderm consists of repeating structural units, thus their structures are relatively more regular [19,20,21,22]. Factually, there are great differences among the structures of FS from various sea cucumbers, due to the diversity in chain lengths of repeating units, glycosidic linkages and/or sulfation patterns [5,22,23,24]. It is reported that FS also has multiple bioactivities, such as anti-thrombosis, antivirus, antitumor, anti-inflammation and anticoagulant activity [25,26,27,28]. It is generally considered that FS showed weaker anticoagulant activities, compared with FG, according to the previous studies [28,29]. However, the structure-activity relationship for anticoagulant activity of FS remains unclear, such as the effects of glycosidic linkages and sulfated patterns on its bioactivities.

Apart from FG and FS, a neutral glycan (NG) was also discovered from some sea cucumbers. It showed no significant anticoagulant activity, compared with the sulfated polysaccharides [29].

In this work, three types of polysaccharides, a fucosylated glycosaminoglycan (PmFG), a fucan sulfate (PmFS) and glycogen-like neutral glycans (PmNG-1 & PmNG-2) were isolated and purified from *Pattalus mollis*. For structure and activity analysis, PmFG was depolymerized by H_2_O_2_ treatment and dPmFG-I – -III were fractioned from the depolymerized products. Composition analysis of PmFG and spectral analysis of dPmFG indicated that PmFG comprised a CS-E-like backbone and the branches of l-FucS (including Fuc_2S4S_, Fuc_3S4S_ and Fuc_4S_ with a molar ratio of 2:2.5:1) which linked to the GlcA of the backbone as side chain via α-1,3 glycosidic bonds.

1D/2D NMR spectra of PmFS showed that it had a backbone consisting of repetitively linked {-4-l-Fuc_2S_-α1-}, which was similar to the FS from *Thelenota ananas* [5], while has unique sulfated Fuc branches linked to the backbone via α-1,3 glycosidic bonds. The weight-average molecular mass (Mw, 6.12 kDa) of PmFS was much lower than Mw (61.2 kDa) of the FS from *T. ananas*. In addition, the structures of two neutral glycans, PmNG-1 & -2, were similar to animal glycogen consisting of d-Glc residues which linked via α-1,4 (major) and α-1,6 (minor, branching) glycosidic bonds [29].

Moreover, the anticoagulant activity of PmFG and PmFS was investigated and their structure-activity relationships were discussed. Both of them exhibited potent activity in APTT prolongation and FXase inhibition. Interestingly, compared with some FS from other species of sea cucumber, PmFS showed relatively potent anticoagulant and anti-FXase activities, which might be due to its distinct structure of FucS branches.

## 2. Results and Discussion

### 2.1. Isolation and Purification of Polysaccharides from P. mollis

**Figure 1:**
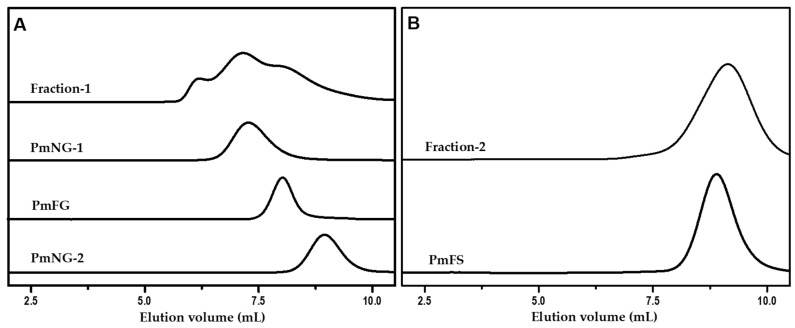
HPGPC profiles of Fraction-1, PmNG-1, PmNG-2 and PmFG (**A**); Fraction-2 and PmFS (**B**). The samples were analyzed on an Agilent Technologies 1200 series equipped with a Shodex OH-pak SB-804 HQ column and eluted with 0.1 M NaCl solution at a flow rate of 0.5 mL/min.

Crude polysaccharides were isolated from *P. mollis* by ethanol precipitation after papain enzymolysis, as described in literature [29,30,31]. The crude polysaccharides were then fractioned into two fractions, Fraction-1 and Fraction-2, by the addition of ethanol at the final concentration of 40% and 60% (*v*/*v*), respectively. PmFG, PmNG-1 and PmNG-2 were further isolated from Fraction-1 by ethanol fractionated precipitation (at the presence of 0.5 M KOAc) and strong anion-exchange (FPA98) chromatography. PmFS was obtained from Fraction-2 by strong anion-exchange (FPA98) chromatography and Sephadex G-100 (1.5 cm × 150 cm) chromatography. The purities of these samples were detected by the high-performance gel permeation chromatography (HPGPC) using an Agilent technologies 1200 series apparatus (Agilent Co., USA) equipped with a Shodex OH-pak SB-804 HQ column (8 mm × 300 mm) and RID and DAD detectors, as described previously [10,30]. The single symmetric peak of each sample in the HPGPC profile (Figure 1) indicated that each sample was a homogenous polysaccharide. Additionally, no absorption observed at 280 or 260 nm indicated the absence of protein or nucleic acids.

### 2.2. Physicochemical Analysis

**Figure 2:**
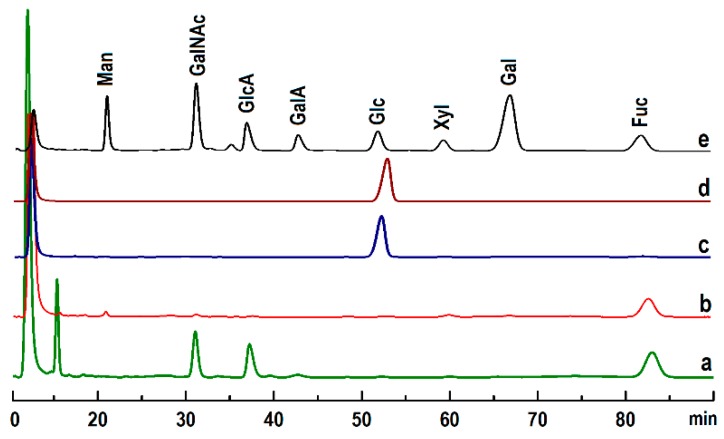
HPLC profiles of monosaccharide-PMP derivates of PmFG (**a**), PmFS (**b**), PmNG-2 (**c**), PmNG-1 (**d**) and standard monosaccharides (**e**).

The monosaccharide compositions of polysaccharides were analyzed by reverse-phase HPLC according to PMP derivatization procedures [30,32]. The results showed that PmFG was composed of three monosaccharides, GlcA, GalNAc and Fuc (Figure 2), while PmFS contained only Fuc, and PmNG-1 and PmNG-2 contained only the monosaccharide of Glc.

**Figure 3:**
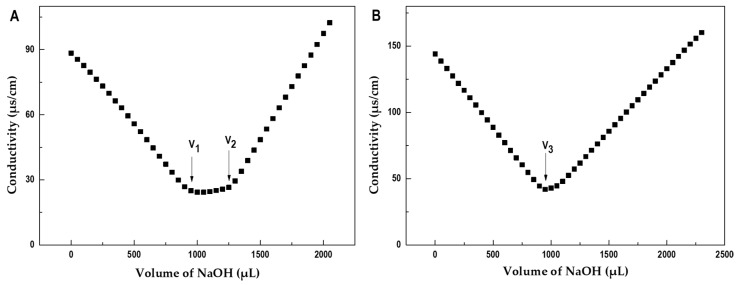
Conductimetric titration curves of PmFG (**A**) and PmFS (**B**).

The acidic groups in PmFG and PmFS were determined by a conductimetric method [30]. Data for the SO_3_^−^ and COO^−^ determinations are shown in Figure 3 and Table 1. In the titration curve of PmFG, two inflection points *V*_1_ and *V*_2_ indicated it contained two types of acidic groups, which was consistent with that of other FG reported previously [28,29,30]. While in the titration curve of PmFS, only one inflection point *V*_3_ was detected, in accordance with that it contained the only kind of acidic group, sulfate. The contents of the sulfate groups of PmFG and PmFS were both 30.4%. The SO_3_^−^/COO^−^ molar ratio in PmFG was estimated to be 3.2, and the sulfate/Fuc molar ratio in PmFS was about 0.91.

The optical rotation of these polysaccharides was detected and is shown in Table 1. Under certain wavelength and temperature conditions, the optical phenomenon of an optically active substance reflects the specific structure and specific rotation along with the structure changes. The specific rotations of PmFG (−75.8°) and PmFS (−115.2°) were both levorotatory, which was compatible with the residues of α-l-Fuc [33], while the specific rotations of PmNG-1 (+172.4°) and PmNG-2 (+140.3°) were both dextrorotatory, which was consistent with α-d-glucose [29].

Moreover, the Mw values of these native polysaccharides were determined by HPGPC using a Shodex 804-HQ column (Table 1). The Mw of PmFG was 60.3 kDa, which was similar to that of FG from other sea cucumbers [8,28,29,30]. Notably, the Mw of PmFS (6.12 kDa) was obviously lower than that of the FS from other species, which varied from tens to hundreds of kDa [5,24,28,31]. Additionally, the Mws of the two NGs were estimated to be 275.6 kDa (PmNG-1) and 22.5 kDa (PmNG-2), respectively, which were consistent with the chromatographic behavior in their HPGPC profiles.

**Figure 4:**
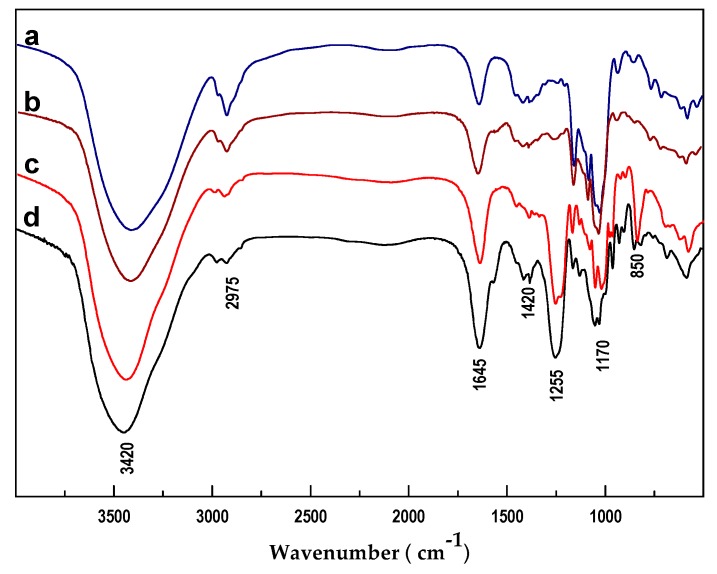
FT-IR spectra of PmNG-1 (**a**), PmNG-2 (**b**), PmFS (**c**) and PmFG (**d**).

The functional groups of these polysaccharides were analyzed by IR spectra (Figure 4). In the four spectra, the broad signals at 3250–3750 cm^−1^ and 2975 cm^−1^ were from the stretching vibrations of O-H and C-H, respectively [32,34]. The signals at 1020–1070 cm^−1^ were assigned to the stretching vibration of C-O-C in the polysaccharide skeleton [28]. The signal peaks of 1255 and 850 cm^−1^ in both PmFG and PmFS spectra were derived from the stretching vibrations of S=O and C-O-S in sulfate esters, indicating that the two polysaccharides were substituted by sulfate groups [29]. Additionally, the strong signal peak of PmFG at 1645 cm^−1^ was generated by the stretching vibration of C=O in GalNAc and GlcA. The band at 1420 cm^−1^ came from the symmetric stretch vibration of COO^−^ in GlcA [10,28,34]. The results showed that different kinds of polysaccharides had their own characteristic signals in FT-IR spectra.

### 2.3. NMR Analysis

**Figure 5:**
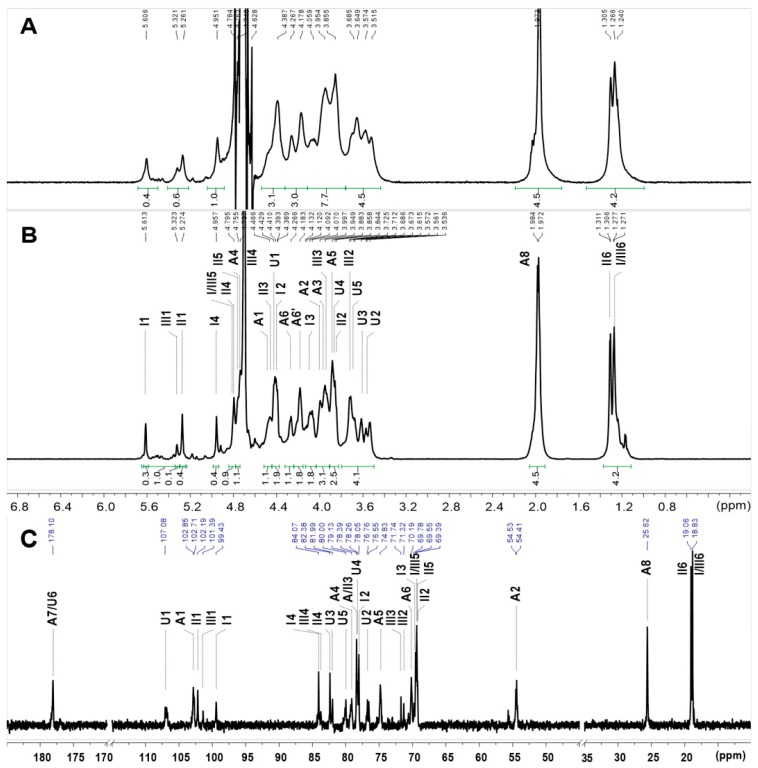
^1^H (**A**,**B**) and ^13^C (**C**) NMR spectra of PmFG (**A**) and dPmFG-II (**B**,**C**) and signal assignments. I, Fuc_2S4S_; II, Fuc_3S4S_; III, Fuc_4S_; U, GlcA; A, GalNAc.

The structural features of the sulfated polysaccharides were further elucidated by NMR spectral analysis. For PmFG, in the ^1^H NMR spectra (Figure 5A), the signals observed in the region approximating 5.2–5.7 ppm could be assigned to anomeric protons of α-l-Fuc residues with different sulfation patterns, including 2,4-di-O-sulfated (Fuc_2S4S_, 5.61 ppm), 3,4-di-O-sulfated (Fuc_3S4S_, 5.26 ppm) and 4-O-sulfated (Fuc_4S_, 5.32 ppm) with a molar ratio of 2:2.5:1, according to previous studies [11,28]. The upfield signals at ~1.25 and 1.97 ppm were assigned to the distinctive methyl protons of Fuc and GalNAc, respectively. The integral ratio of the two signals was approximately 1:1, indicating that Fuc and GalNAc are equal in mole content [10]. Apart from these characteristic signal peaks, others especially in the region of 3.40–4.80 ppm were broad and overlapped, thus hindering the elucidation of the precise structure of PmFG.

**Figure 6:**
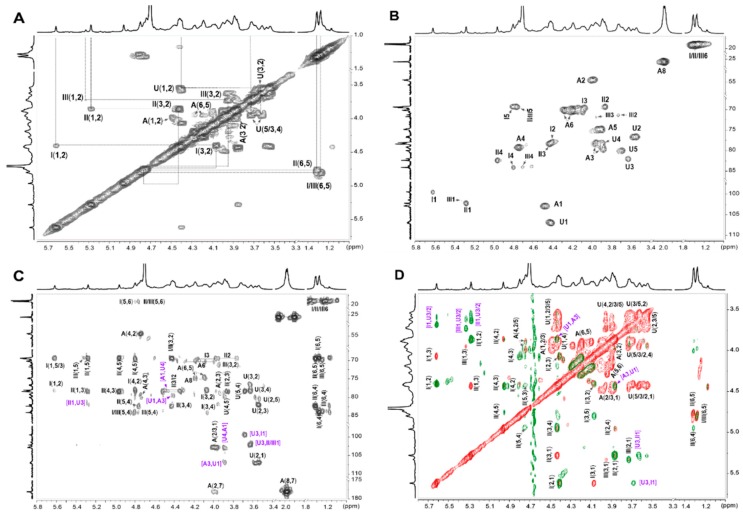
^1^H-^1^H COSY (**A**), ^1^H-^13^C HSQC (**B**)/HMBC (**C**), and superimposed ^1^H-^1^H TOCSY (red)/ROESY (green) (**D**) and signal assignments (purple: the correlation signals of glycosidic bonds). I, Fuc_2S4S_; II, Fuc_3S4S_; III, Fuc_4S_; U, GlcA; A, GalNAc.

To further study the precise structure, its depolymerized product, dPmFG, was prepared by H_2_O_2_ in the presence of cupric ion as catalyst. The dPmFG was further fractioned to three fractions, dPmFG-I – -III, by GPC using Sephadex G-100 column, among which, dPmFG-II was subjected to spectra analysis to obtain the structural data of PmFG. Its signals in ^1^H NMR spectrum were similar with that of PmFG but obviously more explicit (Figure 5A,B). Its ^13^C NMR (Figure 5C) and 2D NMR (^1^H-^1^H COSY/TOCSY/ROESY and ^1^H-^13^C HSQC/HMBC) were also recorded (Figure 6). In the ^1^H NMR spectrum of dPmFG-II, the signals at ~1.25 and 1.98 ppm could also be readily assigned to the methyl protons of Fuc residues (-CH_3_) (Figure 5B), and the signals at 5.61 ppm, 5.27 ppm and 5.32 ppm were ascribed to the anomeric protons of three types of α-l-Fuc residues [11,28]. From these signals, the intra-residue signals in the three types of *α*-l-Fuc were determined from the ^1^H-^1^H COSY and TOCSY spectra (Figure 6A,D). The downfield shifts at 4.42/77.9 ppm and 4.80/84.1 ppm, 4.45/78.4 ppm and 4.96/82.4 ppm and 4.70/83.7 ppm indicated that the sulfate substitutions were at 2,4-, 3,4- and 4-positions, respectively [28]. Additionally, the spin-spin coupling systems from GlcA (U) and GalNAc (A) were also observed based on the COSY and TOCSY spectra. Moreover, the carbon signals were assigned based on the resonance signals of protons in ^1^H-^13^C HSQC spectrum (Figure 6B). The signals at 4.73/79.1 ppm and 4.27 & 4.10/70.2 ppm in the residue GalNAc indicated that *O-4* and *O-6* were both substituted by sulfate esters [11].

The sequence and linkages of these residues were confirmed by the signal correlations in the ^1^H-^13^C HMBC and ^1^H-^1^H ROESY spectra (Figure 6C,D). Specifically, GlcA and GalNAc residues were linked with alternating β-1,3 and β-1,4 bonds according to the cross-peak of 3.97 ppm (H-3, GalNAc) and 4.40 ppm (H-1, GlcA), the correlation of 3.86 ppm (H-4, GlcA) and 4.48 ppm (H-1, GalNAc) in the ROESY spectrum [10]. The linkages were reconfirmed by the correlations of H-1 (GalNAc) and C-4 (79.2 ppm, GlcA), and H-1 (GlcA) and C-3 (76.7 ppm, GalNAc) in the HMBC spectrum. Additionally, three types of α-l-Fuc residues were linked to *O-3* of GlcA as side chains according to the correlations of H/C-1 (Fuc) and H/C-3 (GlcA) in the ROESY and HMBC spectra [28,30].

Based on the above analysis, the structure of dPmFG-II was deduced. The chemical shift assignments were shown in Table 2. Its backbone sequence was {-4-d-GlcA-β-1,3-d-GalNAc_4S6S_-β-1-}, the same as that of CS-E. The mono-l-Fuc side chains including three types (Fuc_2S4S_, Fuc_3S4S_ and Fuc_4S_) were linked to GlcA via α-1,3 glycosidic bonds. Finally, the structure of native PmFG was proposed to be {-[l-Fuc_R_-α-1,3]-d-GlcA-β-1,3-d-GalNAc_4S6S_-β-1,4-}*_n_*, in which R was 2S4S: 3S4S: 4S with a molar ratio of 2:2.5:1.

**Figure 7:**
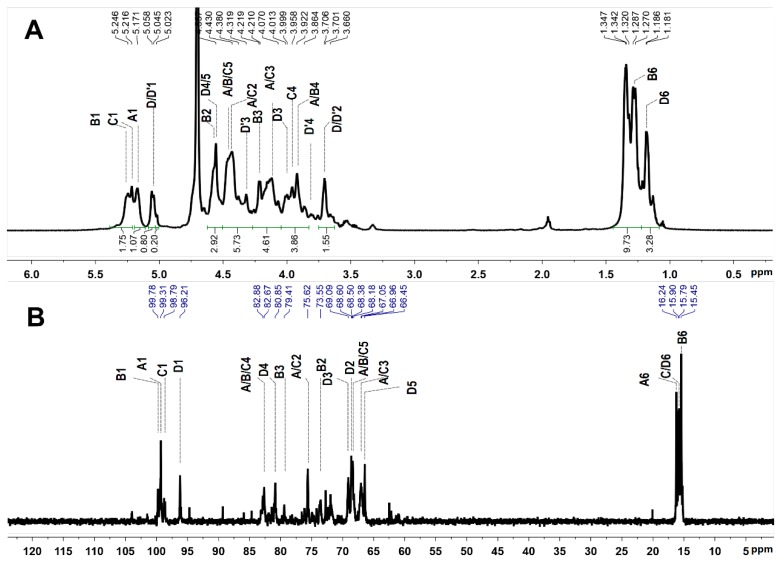
^1^H (**A**) and ^13^C (**B**) NMR spectra of PmFS and signal assignments.

**Figure 8:**
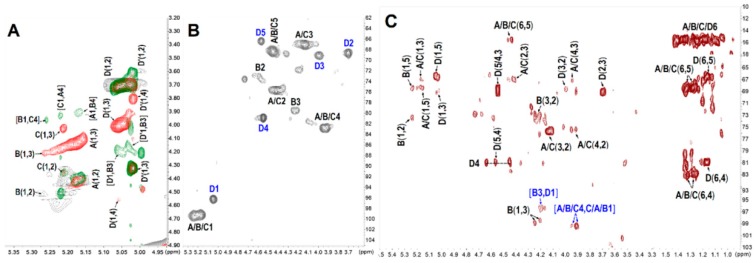
Superimposed ^1^H-^1^H COSY (gray) -TOCSY (red) -ROESY (green) (**A**), ^1^H-^13^C HSQC (**B**) and ^1^H-^13^C HMBC (**C**) spectra of PmFS.

The structure of PmFS was also elucidated by the detailed analysis of its 1D/2D NMR spectra (Figure 7 and Figure 8). In the ^1^H NMR spectrum (Figure 7A), the signals at 1.18–1.35 ppm could be readily assigned to the methyl protons (-CH_3_) of Fuc residues [5,31]. Five signals observed in the downfield 5.0–5.5 ppm region were attributed to the anomeric protons of α-l-Fuc residues. Starting from the resonances, five spin-spin coupling systems (marked as residue A, B, C, D and D’, respectively) were assigned according to the ^1^H-^1^H COSY and TOCSY spectra (Figure 8A). The chemical shifts of corresponding carbon resonances in the five intra-residues were assigned based on the ^13^C and ^1^H-^13^C HSQC spectra (Figure 7B and Figure 8B). The signals at 95–100 ppm were unambiguously ascribed to the anomeric carbons [5].

The H-2 shift values of residues A, B and C at 4.4–4.5 ppm were obviously shifted downfield compared with those of non-sulfated Fuc residues, indicating the sulfation substitution at *O-2* of these residues [24]. These sulfated patterns were reconfirmed by the C-2 shift values at 73–75 ppm. Likewise, the ^1^H/^13^C shift values of residues D/D’ indicated that they possessed *4-/3-O-*sulfated substitutions. Furthermore, the linkages of these residues were proved by the correlation peaks in its ^1^H-^1^H ROESY and ^1^H-^13^C HMBC spectra (Figure 8A,C). The correlations between H-1 and H-4 of residues A, B and C indicated the presence of α-1,3 glycosidic bonds between them. These linkages were reconfirmed by the correlations of H-4 (residues A, B and C) and C-1 (residues C, A and B). The linkage positions were in agreement with the downfield shifts of C-4 [5,28,31]. Additionally, residues D/D’ were linked to *O-3* of residue B from the ROESY and HMBC NMR data, with a molar ratio of 4:1, according to the anomeric proton integrals of D and D’. Taken together, the structure of PmFS was determined to be {-l-Fuc_2S_-α-1,4-[l-Fuc_R_-α-1,3]-l-Fuc_2S_-α-1,4-l-Fuc_2S_-α-1,4-}*_n_*, where R was 4S: 3S with a molar ratio of 4:1. The chemical shift assignments are shown in Table 3.

**Figure 9:**
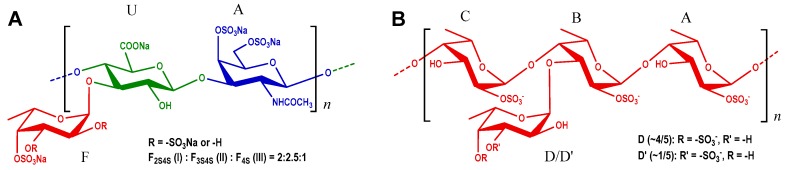
Proposed chemical structures of PmFG (**A**) and PmFS (**B**).

The structure of PmFG and PmFS is shown in Figure 9A,B, respectively.

Interestingly, FS is always reported as a linear polysaccharide with different repeated unit numbers [5,24,31]. Previously, our group also obtained an FS from sea cucumber *T. ananas* with a high regular structure of {-4-l-Fuc_2S_-α-1-}. Compared the two FSs, they have the same backbone of {-4-l-Fuc_2S_-α-1-}, while PmFS is unique in its Fuc branches.

### 2.4. Anticoagulant Activity Evaluation

To assess the anticoagulant activities of the polysaccharides from *P. mollis*, their effects on APTT, PT and TT of normal human plasma were detected compared with LMWH (Table 4).

The results showed that PmFG and PmFS had no significant effect on PT at the concentration up to 128 μg/mL, indicating that they had no effect on the extrinsic coagulation pathway. In the TT assays, PmFG exhibited comparable activity to that of LMWH (the concentration required to double TT were 10.7 μg/mL and 6.06 μg/mL for PmFG and LMWH, respectively), while all the other compounds exhibited no obvious effect on TT at the concentration up to 128 μg/mL [10,29].

The APTT prolonging activity of PmFG was much stronger than that of LMWH (the concentration required to double APTT was 3.50 μg/mL for PmFG and 11.6 μg/mL for LMWH). The concentrations of dPmFG-I – -III required to double APTT were increased from 6.24 μg/mL to 19.3 μg/mL along with the decrease of the molecular weight from 12.8 kDa to 3.71 kDa. This indicated that PmFG and dPmFGs exhibited intrinsic coagulation pathway inhibition activity, and the potency was related to their molecular weight.

The concentrations of PmFS and PmFS-I – -III required to double APTT were 24.3 μg/mL, 22.7 μg/mL, 21.2 μg/mL and 22.5 μg/mL, respectively. Although their APTT prolonging activity was weaker than that of PmFG and dPmFGs, they also had significant anticoagulant activity (their concentrations required for 2APTT were about 2-fold of that of LMWH). Moreover, the activities of PmFSs were similar to TaFS from *T. ananas* (21.7 μg/mL for 2APTT) [5]. Notably, PmFS had the similar backbone to TaFS, but branched with a sulfated α-l-Fuc, and its Mw (6.12 kDa) was only 1/10 of that of TaFS (61.2 kDa). Additionally, the Mw of PmFS-III (5.06 kDa) was approximate to dTaFS (5.14 kDa), while the APTT prolonging activity of PmFS-III was about 3-fold of that of dTaFS (the concentration required for 2APTT was 22.5 μg/mL for PmFS-III and 79.5 μg/mL for dTaFS, respectively) [5]. Compared with some other FS [5,28,31], PmFSs also showed relatively stronger anticoagulant activity.

To further study the anticoagulant mechanism of these polysaccharides, anti-factor IIa and anti-factor Xa activities in the presence of antithrombin (AT) and intrinsic FXase inhibition activity were detected. These sulfated polysaccharides exhibited no significant anti-factor IIa and anti-factor Xa activities in the presence of AT compared with LMWH, suggesting that their anticoagulant targets may be different from the heparin-like compounds [8,10].

Particularly, PmFG displayed potent anti-FXase activity (IC_50_, 13.7 ng/mL), similar to the FG from other sea cucumbers. [10,11,28]. The IC_50_ values of dPmFG-I, dPmFG-II and dPmFG-III for FXase inhibition were 14.0 ng/mL, 17.6 ng/mL and 126 ng/mL, respectively, indicating the decrease of activity with the reduction of the chain length. By contrast, the effect of PmFS (IC_50_, 74.0 ng/mL) was much weaker than PmFG (IC_50_, 13.7 ng/mL) while comparable to LMWH (IC_50_, 59.0 ng/mL). The similar activities of PmFS-I – -III (IC_50_ were 87.9 ng/mL, 109 ng/mL and 99.2 ng/mL, respectively) indicated their activity-chain length relationship may be different from dPmFGs. Interestingly, compared the anti-FXase activity of PmFS-III and dTaFG which have the approximate molecular weight, the former was about 7.5-fold stronger than the latter.

According to the chemical structure, PmFS had a similar backbone to dTaFS, while it is uniquely branched with mono-α-l-Fuc linked via α-1,3 glycocidic bond. Intriguingly, PmFG also contained such Fuc branches, and the FG removal of side chains had no significant anticoagulant activity [35]. Besides, the backbone of PmFS was different from PmFG, and the sulfation pattern and distribution of Fuc branches in PmFG and PmFS were also different. PmFG was branched with a sulfated α-l-Fuc in every disaccharide unit, while PmFS possessed a sulfated α-l-Fuc in every trisaccharide unit; and the branch substitutes of PmFG were mainly di-O-sulfated α-l-Fuc, while those of PmFS were mono-O-sulfated α-l-Fuc. The distinctive branch structure of PmFS might contribute to its relatively more potent anticoagulant action compared with FS from other sea cucumbers, and the study of the structure-activity relationship of PmFG and PmFS could provide valuable data to further develop the novel FXase inhibitors.

## 3. Materials and Methods

### 3.1. Materials

Dried sea cucumber *P. mollis* was purchased from Guangzhou, China. Amberlite FPA98 anion-exchange resin was obtained from the Rohm and Haas Company (St. Louis, MO, USA). Deuterium oxide (D_2_O, 99.9% Atom D) was obtained from Sigma-Aldrich (Shanghai, China). LMWH (Enoxaparin, 0.4 mL × 4000 AXaIU) was obtained from Sanofi-Aventis (Paris, France). The activated partial thromboplastin time (APTT), prothrombin time (PT) and thrombin time (TT) reagents, and standard human plasma were obtained from Teco Medical (Neufahrn N.B., Germany). Both Biophen FVIII: C kit and Biophen Heparin Anti-IIa/Anti-Xa kits were obtained from Hyphen Biomed (Paris, France). Human factor VIII was from Bayer HealthCare LLC (Berlin, Germany). All other chemicals were of reagent grade and are commercially available.

### 3.2. Isolation and Purification of Polysaccharides

Crude polysaccharides were extracted from the body wall of the sea cucumber *P. mollis* according to the method described previously [24,29,30]. Briefly, 300 g dried body wall of *P. mollis* was treated with papain (EC 3.4.22.2), followed by treatment with 0.5 M sodium hydroxide. After neutralization, the mixture was centrifuged at 4000 rpm × 15 min to remove the residues. The supernatant was precipitated by addition of ethanol [final concentration of 40% and 60% (*v*/*v*)] and centrifuged. The two precipitates obtained were designated as Fraction-1 and Fraction-2.

Fraction-1 was decolorized with 3% H_2_O_2_ at 45 °C for 2 h (pH 10) according to our previous method [30,31]. The solution was then treated with ethanol at a final concentration of 40% in the presence of KOAc (0.5 M), followed by centrifugation. The precipitate (A) and supernatant (B) were collected. The precipitate (A) was further purified with strong FPA98 ion-exchange chromatography and sequentially eluted with H_2_O, 0.5 M, 1.0 M, 1.5 M, 2.0 M and 3.0 M NaCl aqueous solution. The 1.5 M NaCl eluate was dialyzed by ultrafiltration with a 3 kDa molecular weight cut-off membrane (Spectrum Laboratories Inc., Piscataway, NJ, USA) and lyophilized to yielded PmFG. The fraction eluted with H_2_O was collected and further purified by ethanol precipitation (40%, *v*/*v*) in the presence of KOAc (0.5 M). After centrifugation, the supernatant was dialyzed and lyophilized to furnish PmNG-1. Additionally, the supernatant (B) was purified by ethanol precipitation with a final concentration 60%. After centrifugation, the precipitate was lyophilized to give PmNG-2.

Fraction-2 was decolorized using the same method mentioned above. The mixture was also treated with a final concentration of 60% ethanol and followed by centrifugation. The precipitate was collected and further purified by FPA98 ion-exchange chromatography eluted using H_2_O, 0.5 M, 1.0 M, 1.5 M, 2.0 M and 3.0 M NaCl aqueous solution as eluents. The 2.0 M NaCl fraction was dialyzed by ultrafiltration with a 3 kDa molecular weight cut-off membrane and lyophilized. The ^1^H NMR spectrum of this fraction showed that it contained some trace amino sugars. Thus, it was further purified by ion-exchange chromatography using a DE-52 column (3.0 cm × 18 cm, GE Healthcare, Uppsala, Sweden) to yield PmFS.

The purity of these polysaccharides was checked by the HPGPC using an Agilent technologies 1200 or 1260 series apparatus (Agilent Co., Santa Clara, CA, USA) equipped with a Shodex OH-pak SB-804 HQ column (8 mm × 300 mm) and RID and DAD. Chromatographic conditions and procedures were performed according to the established method [10,29].

### 3.3. Analysis of Physicochemical Properties

The optical rotation was measured on the autopol VI, Rudolph research analytical, USA. The sulfate/carboxyl ratio or sulfate group content of PmFG and PmFS was determined by a classic conductimetric method [29].

The Mw values of these polysaccharides were estimated by HPGPC using a Shodex OH-pak SB-804 HQ column (8 mm × 300 mm). Chromatographic conditions and procedures were performed according to the previous method [29]. A standard curve was established by standard D-series Dextrans (D 2–8) and corrected by five reference FG (low molecular weight FG with Mw 27.76 kDa, 13.92 kDa, 8.238 kDa, 5.279 kDa and 3.118 kDa) or by an FS with known molecular weight of 2.5 kDa. Molecular weight calculations were performed by a GPC software, version B01.01 (Agilent Co., Santa Clara, CA, USA).

Monosaccharide compositions of PmFG, PmFS, PmNG-1 and PmNG-2 were determined by HPLC after strong acid hydrolysis and derivatization with PMP according to the procedures in our previous reports [30,32]. Each polysaccharide (2 mg) was dissolved in 2 M trifluoroacetic acid (TFA), then the vessel was sealed and incubated at 110 °C for 4 h in a heating block. Each reaction mixture was then evaporated to remove residual TFA with methanol five times, after which, the samples were dissolved in 500 μL H_2_O. Then, 100 μL of each sample solution, 200 μL of 0.5 M PMP in methanol and 100 μL of 0.6M NaOH were mixed and left to react at 70 °C for 30 min. After neutralization, 1 mL of CHCl_3_ was added to remove the residual PMP (repeated four times). The top aqueous layer was collected for HPLC analysis.

### 3.4. FT-IR and NMR Spectroscopic Analysis

The FI-IR spectra (KBr pellets) of PmFG, PmFS, PmNG-1 and PmNG-2 (~1 mg) was recorded by Nicolet iS10 (Thermo Fisher, Waltham, MA, USA) in a range of 400–4000 cm^−1^.

NMR spectra were obtained at 298K in deuterium oxide (D_2_O, 99.9% D) by Bruker Avance spectrometer of 600 or 800 MHz, equipped with the ^1^H/^1^^3^C dual probe in FT mode as described previously [11]. All samples were dissolved in D_2_O and lyophilized three times to replace exchangeable protons with D_2_O and then were dissolved in 0.5 mL of 99.9% D_2_O at a concentration of 10–20 g/L. The NMR data were analyzed using MestReNova software version 8.0.

### 3.5. Preparation of dPmFG-I – -III and PmFS-I – -III

As a polysaccharide with a high Mw, PmFG showed broad and overlapped signals in the ^1^H NMR spectrum. To further elucidate its structure and study the structure-activity relationship in detail, its depolymerized product, dPmFG, was prepared by H_2_O_2_ depolymerization according to our previous methods with minor modifications [28,30,36]. PmFG (290 mg) and 2 mg of copper acetate were dissolved in 10.6 mL H_2_O. 3.3 mL 10% H_2_O_2_ solution was added and the mixture was allowed to react at 35 °C for 3 h. Residual H_2_O_2_ was removed by ethanol precipitation (80%, *v*/*v*) and the precipitate was collected by centrifugation (4000 rpm × 10 min) three times to give dPmFG. Then, dPmFG was fractionated by Sephadex G-100 column (1.5 cm × 150 cm) to three fractions, designated as dPmFG-I, II and III, according to the molecular weight difference. When compared with FS, which possesses linear structures as reported in previous literature [5,24,31], PmFS was branched by α-l-Fuc monosaccharide in every trifucose unit. To further study its structure-activity relationship in detail, PmFS was also fractionated by Sephadex G-50 (1.5 cm × 150 cm) chromatography to yield PmFS-I, II and III. Each fraction had a different molecular weight and showed a narrower molecular weight distribution than PmFS.

### 3.6. Anticoagulant Activity Assays

The anticoagulant activity was detected using APTT, PT and TT reagents and standard human plasma on a coagulometer (TECO MC-4000, Neufahrn N.B., Germany) as previously described [10,29].

According to our previous method [5,10], the inhibition of intrinsic FXase was measured using the BIOPHEN FVIII: C kits and recombinant human FVIII. The anti-FIIa and anti-FXa activities in the presence of AT were determined using BIOPHEN Heparin Anti-FIIa kits and Heparin Anti-FXa kits, respectively.

## 4. Conclusions

In this work, two types of sulfated polysaccharides, PmFG and PmFS, were purified from *P. mollis*. Their physicochemical properties and chemical structures were analyzed and characterized. PmFG comprised a CS-like backbone and monosaccharide branches of sulfated L-Fuc (including Fuc_2S4S_, Fuc_3S4S_ and Fuc_4S_ with a molar ratio of 2:2.5:1) linked to the GlcA of the backbone via α-1,3 glycosidic bonds. Particularly, PmFS is structurally distinct from some FS from other sea cucumbers. It has a backbone consisting of repetitively linked {-4-l-Fuc_2S_-α-1-} and unique sulfated Fuc branches (Fuc_4S_ or Fuc_3S_ with a molar ratio of 4:1) linked to the backbone also via α-1,3 glycosidic bonds.

Anticoagulant assays indicated that both PmFG and PmFS possessed strong APTT prolonging activity and intrinsic FXase inhibitory activity. Interestingly, when compared with the FS from other sea cucumbers reported previously, PmFS showed relatively potent anti-FXase activity, which might be attributed to its distinct structure of sulfated Fuc branches. The structures and anti-FXase activity of PmFS and PmFG were compared to further clarify their structure-activity relationship.

## Figures and Tables

**Table 1:** Chemical compositions and physicochemical properties of the polysaccharides from *P. mollis*.

	Monosaccharide Compositions				SO_3_^−^/COO^−^ (Molar Ratios)	SO_3_^−^/Fuc (Molar Ratios)	Mw (kDa)	Specific Rotations
	GlcA	GalNAc	Fuc	Glc				
PmFG	+	+	+	˗	3.2	/	60.3	−75.8°
PmFS	˗	˗	+	˗	/	0.9	6.12	−115.2°
PmNG-1	˗	˗	˗	+	/	/	275.6	+172.4°
PmNG-2	˗	˗	˗	+	/	/	22.5	+140.3

**Table 2:** ^1^H/^13^C NMR chemical shift assignments of dPmFG-II.

Sugar Residues		Chemical Shifts ^a^							
		1	2	3	4	5	6	7	8
U -4)-β-D-GlcA-(1-	H	4.40	3.56	3.69	3.86	3.60	--		
	C	107.1	78.2	80.0	79.2	82.0	178.1		
A -3)-β-D-GalNAc_4S6S_-(1-	H	4.48	4.00	3.97	4.73	3.88	4.27/4.18	--	1.98
	C	102.8	54.5	76.7	79.1	74.8	70.2	178.1	25.6
I α-L-Fuc_2S4S_-(1-	H	5.61	4.42	4.09	4.80	4.81	1.27		
	C	99.4	77.9	69.6	84.1	69.4	18.8		
II α-L-Fuc_3S4S_-(1-	H	5.27	3.84	4.45	4.96	4.76	1.30		
	C	102.2	69.4	78.4	82.4	69.6	19.1		
III α-L-Fuc_4S_-(1-	H	5.32	3.72	3.94	4.70	4.81	1.27		
	C	101.4	71.3	71.7	83.7	69.4	18.8		

^a^ Data were recorded at 298 K in D_2_O with a Bruker Avance spectrometer of 800 MHz; chemical shifts are given in ppm with reference to D_2_O.

**Table 3:** ^1^H/^13^C NMR chemical shift assignments of the PmFS.

Sugar Residues	Chemical Shifts ^a^						
		1	2	3	4	5	6
A	H	5.17	4.43	4.14	3.92	4.44	1.33
	C	99.3	75.6	67.1	82.7	68.5	16.2
B	H	5.25	4.58	4.22	3.92	4.46	1.28
	C	99.8	73.6	79.4	82.9	68.2	15.5
C	H	5.22	4.43	4.16	3.96	4.38	1.35
	C	98.8	75.6	67.0	82.7	68.4	15.9
D	H	5.05	3.70	4.00	4.56	4.56	1.18
	C	96.2	68.6	69.1	80.9	66.5	15.8
D’	H	5.02	3.66	4.32	3.81	--	--
	C	96.2	68.6	69.1	80.9	66.5	15.8

^a^ Data were recorded at 298 K in D_2_O with a Bruker Avance spectrometer of 800 MHz; chemical shifts were given in ppm with reference to D_2_O.

**Table 4:** APTT prolongation and anti-FXase activities of polysaccharides.

Sample	Molecular Weight	APTT		Anti-FXase (IC_50_)	
	(kDa)	(μg/mL)	(μM)	(ng/mL)	(nM)
PmFG	60.3	3.50	0.0580	13.7	0.227
dPmFG-I	12.8	6.24	0.488	14.0	1.09
dPmFG-II	6.97	7.97	1.14	17.6	2.53
dPmFG-III	3.71	19.3	5.20	126	34.0
PmFS	6.12	24.3	3.97	74.0	12.1
PmFS-I	8.64	22.7	2.63	87.9	10.2
PmFS-II	6.23	21.2	3.40	109	17.5
PmFS-III	5.06	22.5	4.45	99.2	19.6
TaFS ^a^	61.2	21.7	--	197	--
dTaFS ^a^	5.14	79.5	--	745	--
LMWH	3.50~5.50	11.6	2.11~3.31	59.0	10.7~16.9

^a^ Data cited from Shang, F.N. et al., 2018 [5].
